# Anaplastic carcinoma of the pancreas diagnosed by endoscopic ultrasound-guided fine-needle aspiration: a case report and review of the literature

**DOI:** 10.1186/s13256-018-1615-1

**Published:** 2018-05-31

**Authors:** Kohei Oka, Ken Inoue, Satoshi Sugino, Taishi Harada, Toshifumi Tsuji, Shingo Nakashima, Takayuki Katayama, Takashi Okuda, Syuichi Kin, Akihiro Nagata, Toshiyuki Komaki, Keizo Kagawa

**Affiliations:** 1 Department of Gastroenterology and Hepatology, Fukuchiyama City Hospital, 231 Atsunaka-cho, Fukuchiyama-city, Kyoto 620-8505 Japan; 2 Department of Surgery, Fukuchiyama City Hospital, 231 Atsunaka-cho, Fukuchiyama-city, Kyoto 620-0056 Japan; 3 Department of Pathology, Fukuchiyama City Hospital, 231 Atsunaka-cho, Fukuchiyama-city, Kyoto 620-0056 Japan

**Keywords:** Anaplastic carcinoma of the pancreas, EUS-FNA, Cystic change

## Abstract

**Background:**

Anaplastic carcinoma of the pancreas is a rare pancreatic neoplasm with a poor prognosis. It is classified as a variant of ductal adenocarcinoma, but the clinical features and treatment of it remain unknown because of its rarity and aggressiveness. Endoscopic ultrasonography and endoscopic ultrasound-guided fine-needle aspiration are useful techniques for the diagnosis of pancreatic tumors with high sensitivity and specificity.

**Case presentation:**

A 72-year-old Japanese woman presented with a diagnosis of acute pancreatitis, and a cystic lesion with slightly high density area was observed by computed tomography in her pancreatic head. In addition, endoscopic ultrasound revealed a heterogeneous lesion. Endoscopic ultrasound-guided fine-needle aspiration showed pleomorphic atypical cells. We diagnosed anaplastic carcinoma of the pancreas. We resected the lesion, and she has shown no sign of recurrence for > 6 months. There are few reports of anaplastic carcinoma of the pancreas diagnosed by endoscopic ultrasound-guided fine-needle aspiration and treated by surgery. Our analysis indicates that anaplastic carcinoma of the pancreas is more likely than typical ductal carcinomas to have cystic lesions with the tumor.

**Conclusions:**

We report a case of anaplastic carcinoma of the pancreas diagnosed by endoscopic ultrasound-guided fine-needle aspiration and subsequently resected with a clear margin. We speculate that anaplastic carcinoma of the pancreas is more likely to have cystic changes than pancreatic ductal adenocarcinoma. When we diagnose pancreas tumor as having cystic changes, anaplastic carcinoma of the pancreas should be considered one of the differential diagnoses.

## Background

Anaplastic carcinoma of the pancreas (ACP) is a rare pancreatic neoplasm with a poor prognosis [1, 2]. It is classified as a variant of ductal adenocarcinoma, but the clinical features and treatment of ACP remain unknown because of its rarity and aggressiveness. Endoscopic ultrasonography (EUS) and endoscopic ultrasound-guided fine-needle aspiration (EUS-FNA) are useful techniques for the diagnosis of pancreatic tumors with high sensitivity and specificity [3, 4]. There are case reports describing ACP, but only a few cases of ACP diagnosed by EUS-FNA have been reported. Here, we report the case of a patient with ACP diagnosed by EUS-FNA who subsequently underwent resection. We also discuss the characteristics of ACP, especially in EUS imaging, in a comparison with pancreatic ductal adenocarcinoma (PDAC).

## Case presentation

**Fig. 1:**
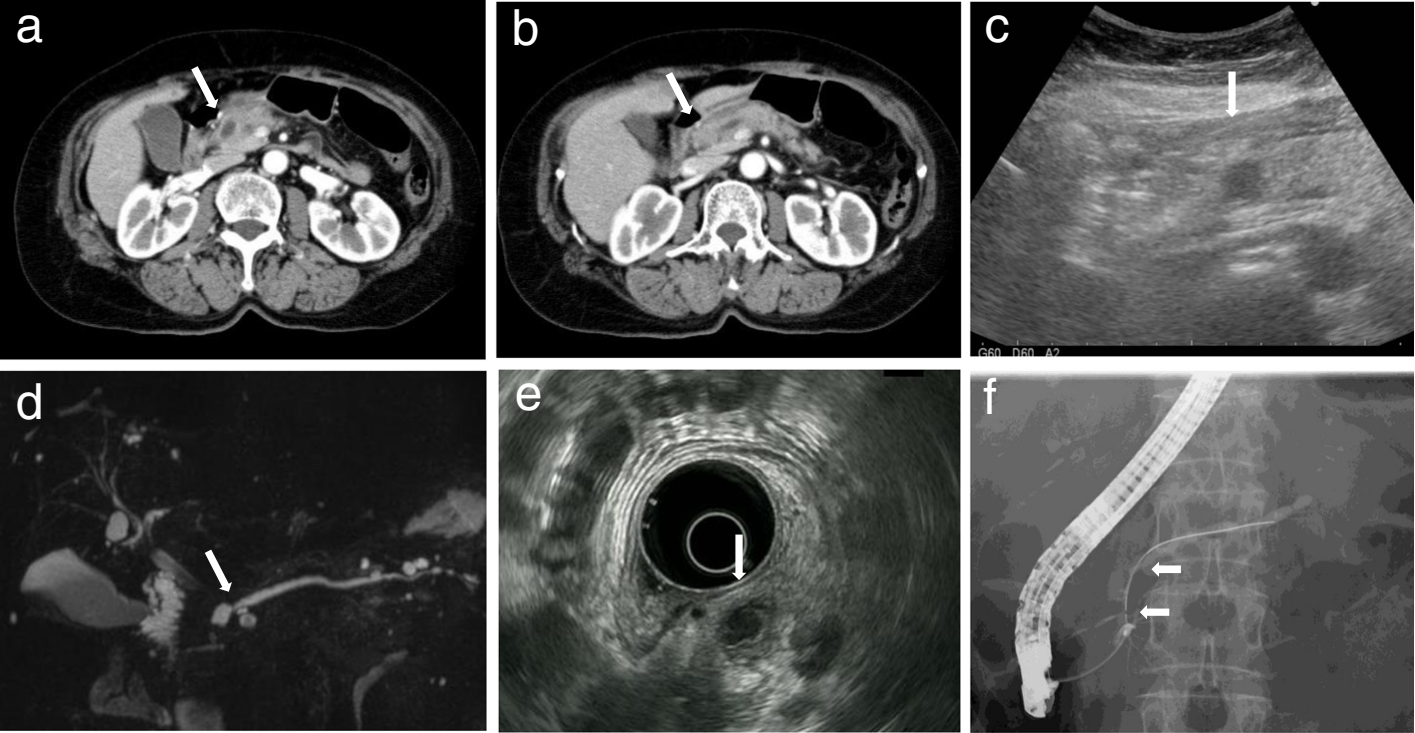
**a**, **b** CT showed a cystic mass in the pancreatic head (arrow). Slight fat stranding suggested that inflammation was localized to the pancreatic head. The MPD was dilated from the body to the tail (arrow). **c** The cystic mass was demonstrated as a hypo-echoic area under US (arrow). **d** MRCP showed that the MPD was obstructed by the cystic mass (arrow). **e** EUS revealed that the masswas 15 mm and comprise of both solid and cystic components (arrow). **f** ERP showed the MPD was obstructed for a 12-mm length (arrow). There was no obvious communication between the cystic mass and the MPD

A 72-year-old Japanese woman presented with complaints of epigastric pain and nausea. The pain had started 3 days before admission and gradually worsened. Laboratory testing showed a high level of serum amylase (AMY) 838 IU/l and pancreatic amylase (P-AMY) 778 IU/l. CA 19-9 was elevated to 86.4 U/ml, but other tumor markers were normal. Computed tomography (CT) scanning showed inflammation localized in the pancreatic head and dilatation of the main pancreatic duct (MPD) (Fig. 1). In addition, a cystic lesion with a slightly high density area was observed by CT in the pancreatic head.

Transabdominal ultrasonography and magnetic resonance cholangiopancreatography (MRCP) were performed. Both ultrasonography and MRCP demonstrated a 14 mm cystic lesion in the pancreatic head. They showed the dilatation of the MPD from the body to the tail of her pancreas. We could not identify a connection between the cystic lesion and the MPD. EUS showed the cystic lesion more clearly than other modalities. EUS revealed that the cystic lesion consisted of both solid and cystic lesions. The solid area was shown as a hypoechoic and heterogeneous tumor, and the cystic area was shown as an anechoic lesion. The EUS also showed that the MPD was dilated to 5 mm, and it was cut off around the mass in the pancreatic head. Endoscopic retrograde cholangiopancreatography (ERCP) showed > 12-mm-long stenosis of the MPD in the pancreatic head. The stenosis prevented a brush for cytology passing the stricture, and it was not possible to obtain a cytology specimen.

**Fig. 2:**
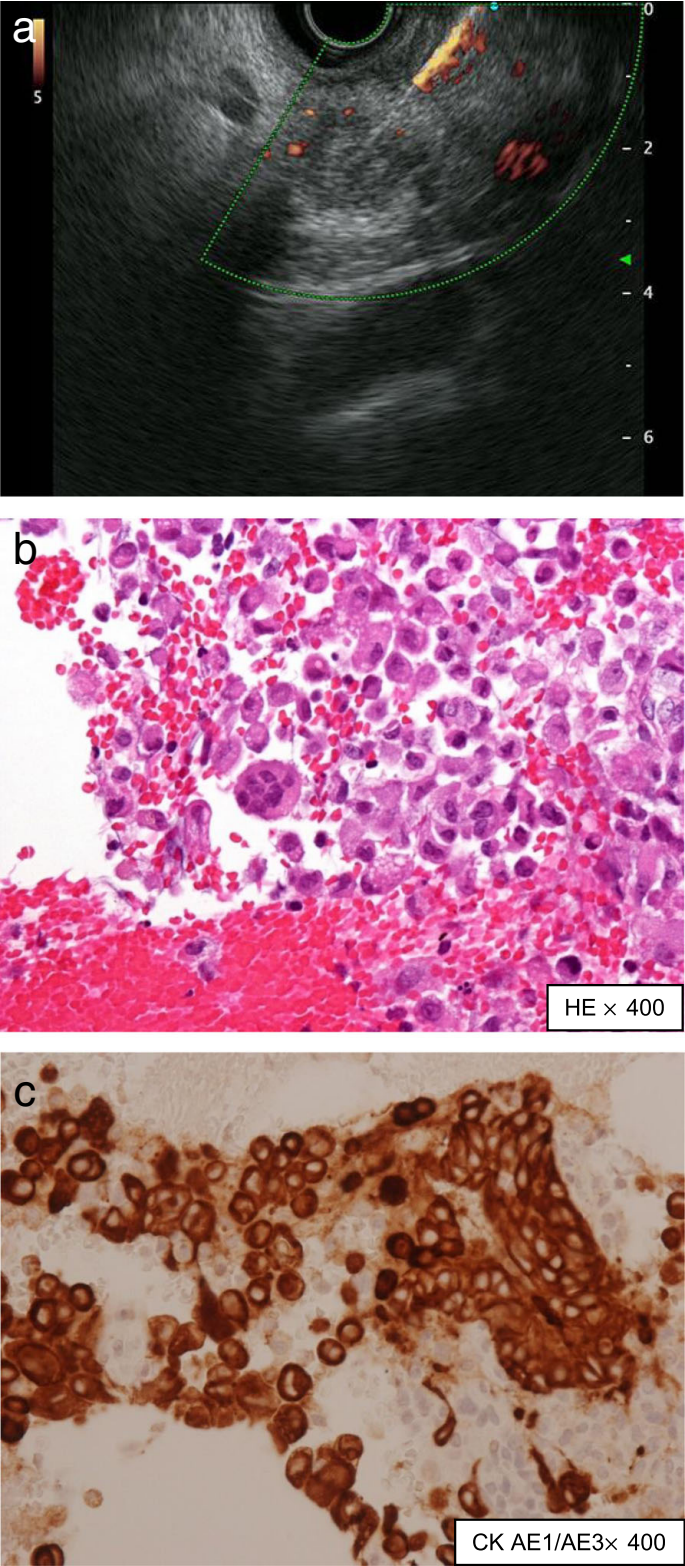
Findings obtained by endoscopic ultrasound-guided fine-needle aspiration. **a** Endoscopic ultrasound-guided fine-needle aspiration was performed to obtain cytology for the solid mass in the pancreatic head. **b** Histology showed pleomorphic large atypical cells (hematoxylin-eosin, magnitude × 400). **c** Cytokeratin AE1/AE3 stain was positive, and thus these cells were epithelial cells (cytokeratin AE1/AE3, magnitude × 400)

We performed EUS-FNA (Fig. 2). An echoendoscope (GF-UCT260, Olympus; Tokyo, Japan) with a 22-gauge needle was used to obtain cytological material: EchoTip ProCore® HD Ultrasound Needle (ECHO-HD-22-C; Cook Medical, USA). We carried out two fine-needle aspiration (FNA) punctures, moving ten times in each puncture. We aspirated the solid area of the tumor, and then the cells were histologically revealed as pleomorphic atypical cells. Immunohistochemical stains were positive for cytokeratin (CK) AE1/AE3 and CK CAM5.2, which confirmed they were epithelial cells. Based on these findings, we diagnosed the tumor as an ACP.

**Fig. 3:**
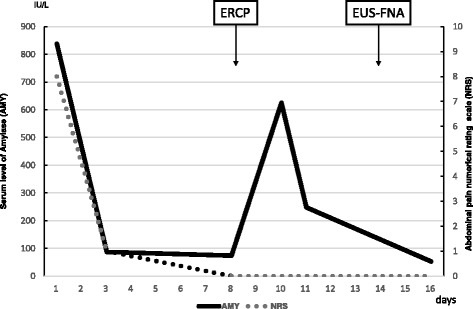
The patient’s clinical course. This graph shows serum level of amylase and 10-point numerical rating scale on abdominal pain. Both of them improved immediately, except for temporary elevation of serum level of amylase after the endoscopic retrograde cholangiopancreatography procedure. *AMY* serum amylase, *ERCP* endoscopic retrograde cholangiopancreatography, *EUS-FNA* endoscopic ultrasound-guided fine-needle aspiration, *NRS* numerical rating scale

**Fig. 4:**
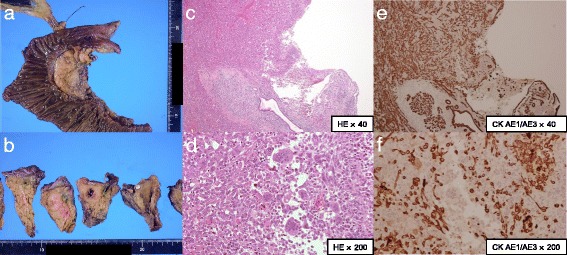
Resected specimen findings. **a** The tumor was not clearly exposed to the surface of the pancreas. **b** The tumor consisted of a yellow nodular mass with the cystic lesion in the center of the mass. The cystic lesion was pathologically a pancreatic duct. A pancreatic calculus was found in this specimen. **c**, **d** Hematoxylin-eosin staining showed spindle cells and multinuclear giant cells, which are characteristic of anaplastic carcinoma of the pancreas. **e**, **f** These cells exhibited immunoreactivity for cytokeratin AE1/AE3. The tumor invaded pancreatic anterior fat tissue

Our patient’s abdominal pain and the elevation of pancreatic enzyme improved (Fig. 3). CT images suggested tumor invasion to the front of her pancreas. Positron emission tomography (PET)-CT and gadolinium-ethoxybenzyl-diethylenetriamine pentaacetic acid (Gd-EOB-DTPA)- enhanced MRI showed no obvious metastasis. We performed a subtotal stomach-preserving pancreaticoduodenectomy, and no major complications occurred. On macroscopic examination, the tumor consisted of a yellow nodular mass with a cystic lesion, as had been shown by EUS. The cystic lesion was pathologically confirmed as a pancreatic duct with some blood pooling. Hematoxylin-eosin staining showed spindle cells, pleomorphic cells, and multinuclear osteoclast-like giant cells (OCGCs), which are characteristic of ACP (Fig. 4).

The resection was completed with a clear margin. The final diagnosis was ACP according to Japan Pancreas Society (JPS) classification, staged pathologically T3(pS+)N0 M0, pStageIIA, according to the Union for International Cancer Control (UICC) TNM staging system. After discharge from our hospital, she began oral tegafur/gimeracil/oteracil (S-1) intake as adjuvant chemotherapy. Due to anorexia and diarrhea as adverse effects, however, the S-1 administration was discontinued after 2 weeks. Although she declined gemcitabine therapy as an alternative, she has shown no evidence of recurrence 6 months after the resection.

## Discussion

ACP is a variant of ductal adenocarcinoma with poor differentiation. It accounts for only 2–7% of all pancreatic adenocarcinomas [1]. It is so aggressive that its median survival time is only 5.7 months (*n* = 18) [2]. Our patient’s ACP showed rapid progression. Although the EUS indicated that the tumor size was 15 mm, it turned out to be 25 mm at the time of surgery at approximately 1 month after the EUS-FNA. This is characteristic of sarcoma-like progression, but ACP is classified as a subtype of ductal tumors because of the presence of ductal carcinoma cells. ACPs are pathologically classified into three variants: pleomorphic type, spindle cell type, and ACP with OCGCs. ACP with OCGCs has an exceptionally better prognosis than the other subtypes.

There are many case reports describing ACP, but only a few cases of ACP diagnosed by EUS-FNA have been reported. We performed a literature search with the terms “Anaplastic carcinoma pancreas” and “EUS-FNA”, using PubMed database for the period from 2006 to September 2016. We found seven reports of 19 cases of ACP during that period diagnosed by EUS-FNA (including our patient; Table 1) [5–11]. In most of the cases, the tumors were shown as hypoechoic and heterogeneous by EUS. The features of their components varied; of the components, 30% (3 of 10) were a fully solid mass and 60% (6 of 10) were mixed with solid and cystic lesions.

**Table 1:** Reported cases of anaplastic carcinoma of the pancreas diagnosed by endoscopic ultrasound-guided fine-needle aspiration

Author	Reference	Age/Sex	sites	Size (mm)	Echogenicity	Homogeneity	Solid or Cystic	Pathology	Treatment
Chopra *et al*.	[6]	89 M	Head	20	Hypoechoic	Heterogeneous	–	OGC	BSC
		64 F	Head	31	Hypoechoic	Heterogeneous	Mixed	OGC	Surgery
Layfield *et al*.	[9]	71 M	Head	–	–	–	–	Pleomorphic	BSC
		81 F	Head	–	–	–	–	Pleomorphic	BSC
		59 M	Head	–	–	–	–	Pleomorphic	BSC
		81 M	Head	–	–	–	–	Pleomorphic	BSC
		59 M	Head	–	–	–	–	OGC	Surgery
		64 M	Body	–	–	–	–	Pleomorphic	BSC
Salla *et al*.	[10]	44 F	Tail	50	Hypoechoic	Heterogeneous	Mixed	Pleomorphic	–
Khashab *et al*.	[8]	60 M	Head	43	Hypoechoic	Heterogeneous	Mixed	–	Chemotherapy
		49 M	Body	58	Hypoechoic	Heterogeneous	Mixed	–	Surgery
		44 M	Tail	41	Hypoechoic	Heterogeneous	Mixed	–	Chemotherapy
		85 M	Head	20	Hypoechoic	Homogeneous	Solid	–	Surgery
		58 M	Body	34	Hypoechoic	Heterogeneous	Solid	–	NAC + surgery
		65 M	Head	100	Hypoechoic	Heterogeneous	Solid	–	BSC
Wakatsuki *et al*.	[11]	58 F	Head	–	–	–	–		Chemotherapy
Ergun *et al*.	[7]	60 M	Head	32	Hypoechoic	Heterogeneous	–		Chemotherapy
Aldaoud *et al*.	[5]	37 F	Body	160	Hypoechoic	Homogeneous	Cystic	Pleomorphic	Surgery + adj
Our case		72 F	Head	25	Hypoechoic	Heterogeneous	Mixed	Unclassifiable	Surgery

Adj adjuvant chemotherapy, BSC best supportive care, F female, M male, NAC Neoadjuvant chemotherapy, OGC osteoclast-like giant cell

It is reported that ACPs are more likely than PDACs to have a cystic appearance [2, 12]. It is assumed that the aggressiveness of ACP may induce cystic change because of central necrosis or degeneration [2]. Although these cystic changes may occur not only in ACP but also PDAC, such a change occurs in < 1% of PDACs [13]. ACPs should thus be considered a differential diagnosis in patients with a pancreatic tumor with a cystic lesion. We can also find cystic degeneration in other pancreatic tumors such as adenosquamous carcinoma, neuroendocrine tumors, solid and pseudopapillary tumors, acinar cell carcinoma, and so on [14–16]. In our case, ACP might have induced cystic change due to central necrosis or degeneration into a pancreatic duct, because the cystic lesion was pathologically a pancreatic duct.

In contrast, CT images of an ACP may show features comparable to those of PDACs. Compared to PDACs, ACPs are more hypervascular tumors [8]. It is reported that ACPs often present as a mass with peripheral contrast enhancement in the portal venous phase [2, 12]. In our patient’s case, a cystic lesion with slightly high density area was observed by CT. EUS showed the cystic lesion more clearly than other modalities. EUS also might be useful for detecting ACP because of the high resolution of images, as has been reported for PDAC. EUS-FNA is useful for making a pathological diagnosis of pancreatic tumors, but there are some risks for dissemination [3, 17].

There is no consensus regarding the optimal treatment for ACPs, due to the lack of data and evidence. It is reported that ACP is rare and aggressive with shorter overall survival times than PDAC [2]. This result suggests that an early diagnosis of ACP should be made for better outcomes. Chemotherapy for ACP is also uncertain. There is a report of a patient with ACP who passed a chemosensitivity test and was treated with paclitaxel [11]. Khashab *et al*. suggested that in light of the hypervascularity of ACPs, investigational therapies with agents such as bevacizumab may improve the outcome of patients with ACP [8]. There is no evidence regarding adjuvant chemotherapy for ACP. We administered S-1 to our patient based on a phase 3 trial for PDAC.

## Conclusions

We report a case of ACP diagnosed by EUS-FNA and subsequently resected with a clear margin. We speculate that ACPs are more likely to have cystic changes than PDAC, and we found that EUS-FNA was a useful technique to detect this characteristic change and make a definite diagnosis. When we diagnose the pancreas tumor as having cystic changes, ACP should be considered one of the differential diagnoses. A further accumulation of cases of ACP must be examined to clarify this disease.

## Abbreviations

ACP: Anaplastic carcinoma of the pancreas
AMY: Serum amylase
CK: Cytokeratin
CT: Computed tomography
ERCP: Endoscopic retrograde cholangiopancreatography
EUS: Endoscopic ultrasonography
EUS-FNA: Endoscopic ultrasound-guided fine-needle aspiration
FNA: Fine-needle aspiration
Gd-EOB-DTPA: Gadolinium-ethoxybenzyl-diethylenetriamine pentaacetic acid
JPS: Japan Pancreas Society
MPD: Main pancreatic duct
MRCP: Magnetic resonance cholangiopancreatography
OCGCs: Osteoclast-like giant cells
P-AMY: Pancreatic amylase
PDAC: Pancreatic ductal adenocarcinoma
PET: Positron emission tomography
S-1: Tegafur/gimeracil/oteracil
UICC: Union for International Cancer Control
